# Supplementary material for: Temperature-related death burden of various neurodegenerative diseases under climate warming: a nationwide modelling study
Source: Nat Commun. 2023 Dec 12;14:8236. doi: 10.1038/s41467-023-44066-5 (PMC10716387; doi:10.1038/s41467-023-44066-5)
Supplement: Supplementary file 1 — Supplementary Information [file 41467_2023_44066_MOESM1_ESM.pdf]

Supplementary Information

Temperature-related death burden of various neurodegenerative diseases under climate warming: a nationwide modelling study

Peng Yin[1,†], Ya Gao[2,†], Renjie Chen[2,†], Wei Liu[1], Cheng He[2], Junwei Hao,[3,4*], Maigeng Zhou[1,*] ,Haidong Kan[2,*]

Affiliations:

[1] National Center for Chronic Noncommunicable Disease Control and Prevention, Chinese Center for Disease Control and Prevention, Beijing, China;

[2] School of Public Health, Key Lab of Public Health Safety of the Ministry of Education, NHC Key Lab of Health Technology Assessment, IRDR ICoE on Risk Interconnectivity and Governance on Weather/Climate Extremes Impact and Public Health, Fudan University, Shanghai, China.

[3] Department of Neurology, Xuanwu Hospital, Capital Medical University, Beijing, China.

[4] National Center for Neurological Disorders, Beijing, China.

† These authors contributed equally to this work: Peng Yin, Ya Gao, Renjie Chen

* Corresponding author: Haidong Kan, Maigeng Zhou, Junwei Hao

Supplementary Table 1. The ICD-10 codes and deaths of various neurodegenerative diseases in China Mainland and different climatic zones from 2013 to 2019.

Disease Names	ICD-10 codes[1]	Nationwide	Climatic zones				
			Subtropical monsoon zone	Temperate monsoon zone	Temperate continental zone	Tropical monsoon zone	Highland alpine zone
Neurodegenerative disease	F01-F03; G12.2; G20; G21; G23.1-3, G23.8&9; G30; G31	437,218	273,820	146,033	12,724	2,101	2,540
Dementia	F01-F03; G23.1-3; G30; G31	375,776	232,765	127,622	11,203	1,872	2,314
Alzheimer disease	G30	65,254	54,396	9,575	926	283	74
Non-Alzheimer dementias	F01-F03; G23.1-3;G31	310,522	178,369	118,047	10,277	1,589	2,240
Parkinson disease	G20; G21; G23.8&9	51,428	35,677	14,228	1,151	200	172

Abbreviations: ICD-10 = the Tenth Revision of the International Classification of Diseases.

Supplementary Table 2. Summary statistics for daily mean temperatures (°C) in various climatic zones of China Mainland during 2013 to 2019.

Climate zones	Mean ± SD	Percentile				
		P2.5	P25	P50	P75	P97.5
Nationwide	13.5 ± 11.3	-12.5	6.1	15.4	22.6	29.5
Subtropical monsoon zone	17.4 ± 8.1	1.5	11.3	18.4	24.0	29.9
Temperate monsoon zone	11.3 ± 12.1	-15.3	2.0	12.8	21.6	29.2
Temperate continental zone	7.5 ± 12.8	-17.3	-2.9	8.9	18.5	27.1
Tropical monsoon zone	24.4 ± 4.1	14.5	21.9	25.3	27.5	30.1
Highland alpine zone	3.7 ± 11.6	-17.9	-4.7	3.8	11.1	27.3

Abbreviations: SD = standard deviation; P2.5: 2.5th percentile; P25: 25th percentile; P50: 50th percentile; P75: 75th percentile; P97.5: 97.5th percentile.

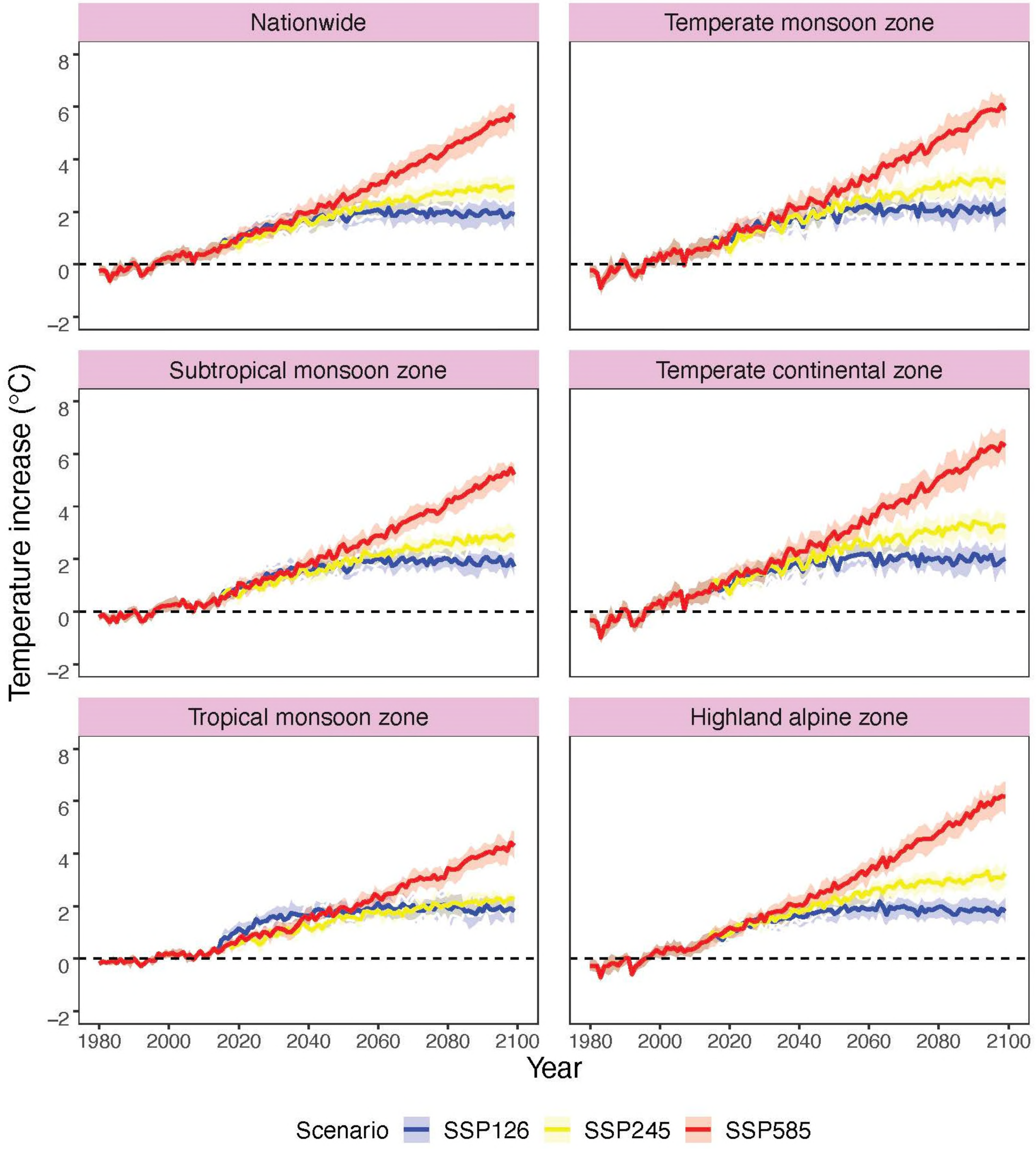

Supplementary Fig. 1. Projected increments in annual mean temperature in 2010-2090 compared with 1980–2009 under climate change scenarios (SSP126, SSP245 and SSP585) in China. Solid lines correspond to the mean annual temperature estimated across the ten GCM-specific modeled series. The shaded area shows its variability, corresponding to the range for each year. SSP = Shared Socioeconomic Pathway. Source data are provided as a Source Data file.

Supplementary Table 3. Projected annual average temperature from GCM-ensemble under climate change scenarios, classified by climatic zones and periods.

Climate zones	Scenarios	1980s-2000s		2030s			2050s			2090s		
		Mean	SD	Mean	SD	Δ	Mean	SD	Δ	Mean	SD	Δ
Nationwide	SSP126	12.1	8.9	13.7	8.9	1.6	14.0	8.9	1.9	14.1	8.9	1.9
	SSP245	12.2	8.9	13.6	8.9	1.5	14.4	8.9	2.2	15.1	8.9	2.9
	SSP585	12.2	8.9	13.8	8.9	1.7	14.9	8.9	2.8	17.6	8.9	5.4
Temperate monsoon zone	SSP126	10.5	11.2	12.1	11.3	1.6	12.6	11.2	2.0	11.2	11.3	2.1
	SSP245	10.6	11.3	12.2	11.3	1.6	13.0	11.2	2.4	11.3	11.3	3.2
	SSP585	10.6	11.2	12.4	11.2	1.9	13.6	11.2	3.0	11.2	11.2	5.8
Subtropical monsoon zone	SSP126	16.8	7.4	18.3	7.4	1.5	18.6	7.3	1.8	18.7	7.2	1.9
	SSP245	16.9	7.5	18.2	7.4	1.3	18.9	7.4	2.0	19.7	7.4	2.8
	SSP585	16.8	7.4	18.4	7.4	1.5	19.4	7.4	2.6	22.0	7.4	5.1

Abbreviations: GCM = General climate models. SSP = Shared Socioeconomic Pathway. Δ denotes the change in the annual average temperatures from baseline period (1980s-2000s).

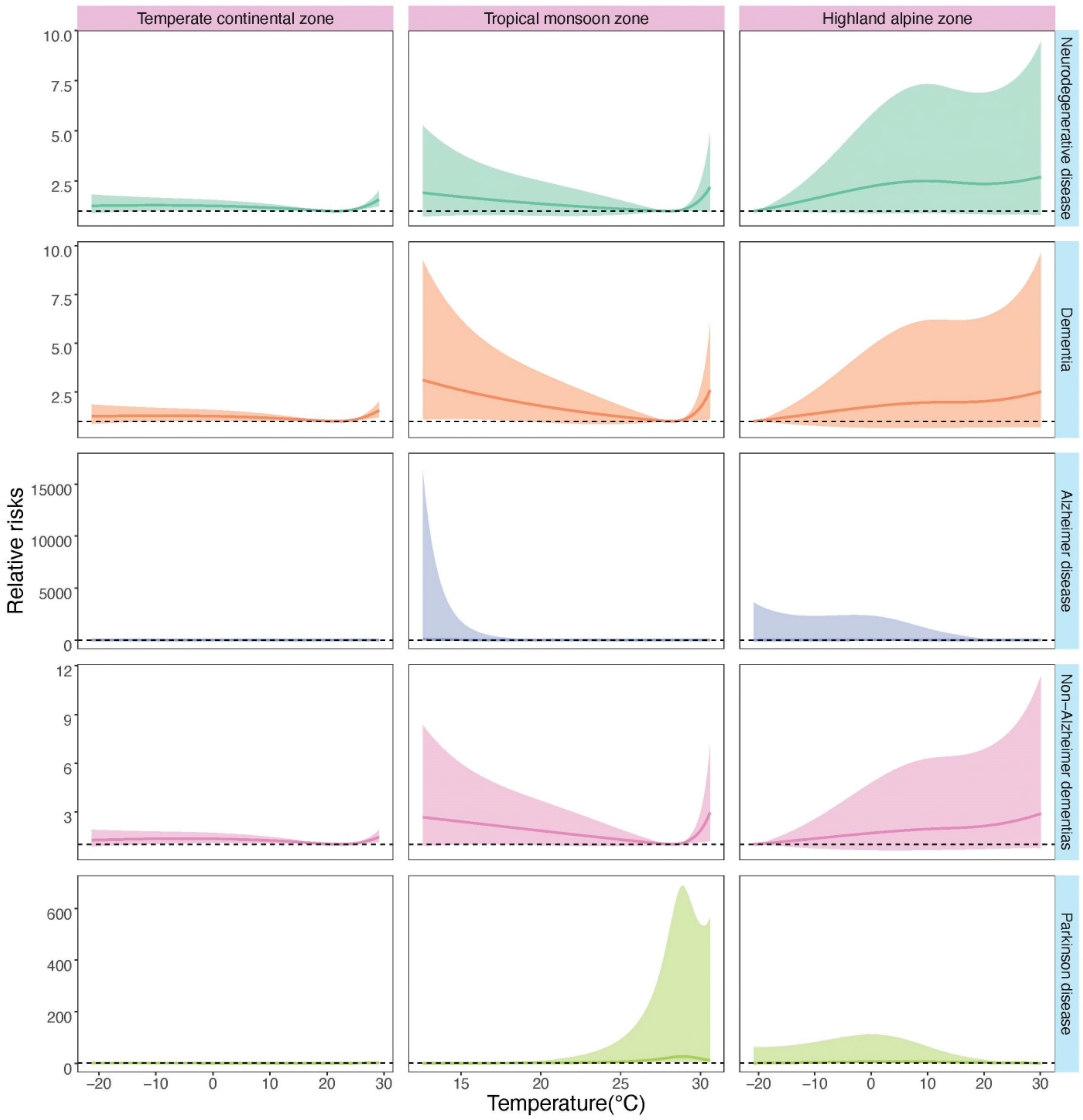

Supplementary Fig. 2. Exposure-response curves for the associations between daily mean temperature and death of overall and specific neurodegenerative diseases in Temperate continental zone ,Tropical monsoon zone, Highland alpine zone of China. The associations were presented as the cumulative relative risks comparing a given temperature to the minimum-mortality temperature over lag 0–14 day. The line represents the point estimates, and the shading indicates corresponding 95% confidence intervals. Source data are provided as a Source Data file.

Subgroup	Sample size (proportion)	Extremely low temperature	Extremely high temperature
**Overall**	**437,218 (100%)**	**1.95 (1.81, 2.10)**	**1.29 (1.26, 1.32)**
**Age**			
<65	34,243 (7.8%)	1.25 (0.98, 1.60)	1.39 (1.26, 1.52)
>=65&<74	60,162 (13.8%)	1.45 (1.20, 1.75)	1.30 (1.21, 1.38)
>=75	342,812 (78.4%)	2.17 (1.99, 2.36)	1.28 (1.25, 1.32)
**Sex**			
Male	203,105 (46.5%)	1.84 (1.66, 2.06)	1.25 (1.21, 1.30)
Female	234,113 (53.5%)	2.06 (1.86, 2.29)	1.33 (1.28, 1.37)
**Education**			
Middle school and below	400,614 (91.6%)	1.99 (1.84, 2.16)	1.30 (1.27, 1.34)
High school and above	34,675 (7.9%)	1.50 (1.18, 1.92)	1.19 (1.07, 1.33)
**Age&Sex**			
<65 & Male	20,615 (4.7%)	1.70 (1.24, 2.35)	1.36 (1.21, 1.53)
>=65&<74 & Male	33,987 (7.8%)	1.39 (1.08, 1.78)	1.34 (1.23, 1.46)
>=75 & Male	148,503 (34.0%)	2.04 (1.79, 2.32)	1.22 (1.17, 1.28)
<65 & Female	13,628 (3.1%)	1.00 (0.96, 1.04)	1.85 (1.23, 2.78)
>=65&<74 & Female	26,175 (6.0%)	1.52 (1.14, 2.03)	1.25 (1.13, 1.38)
>=75 & Female	194,309 (44.4%)	2.29 (2.03, 2.57)	1.33 (1.28, 1.38)

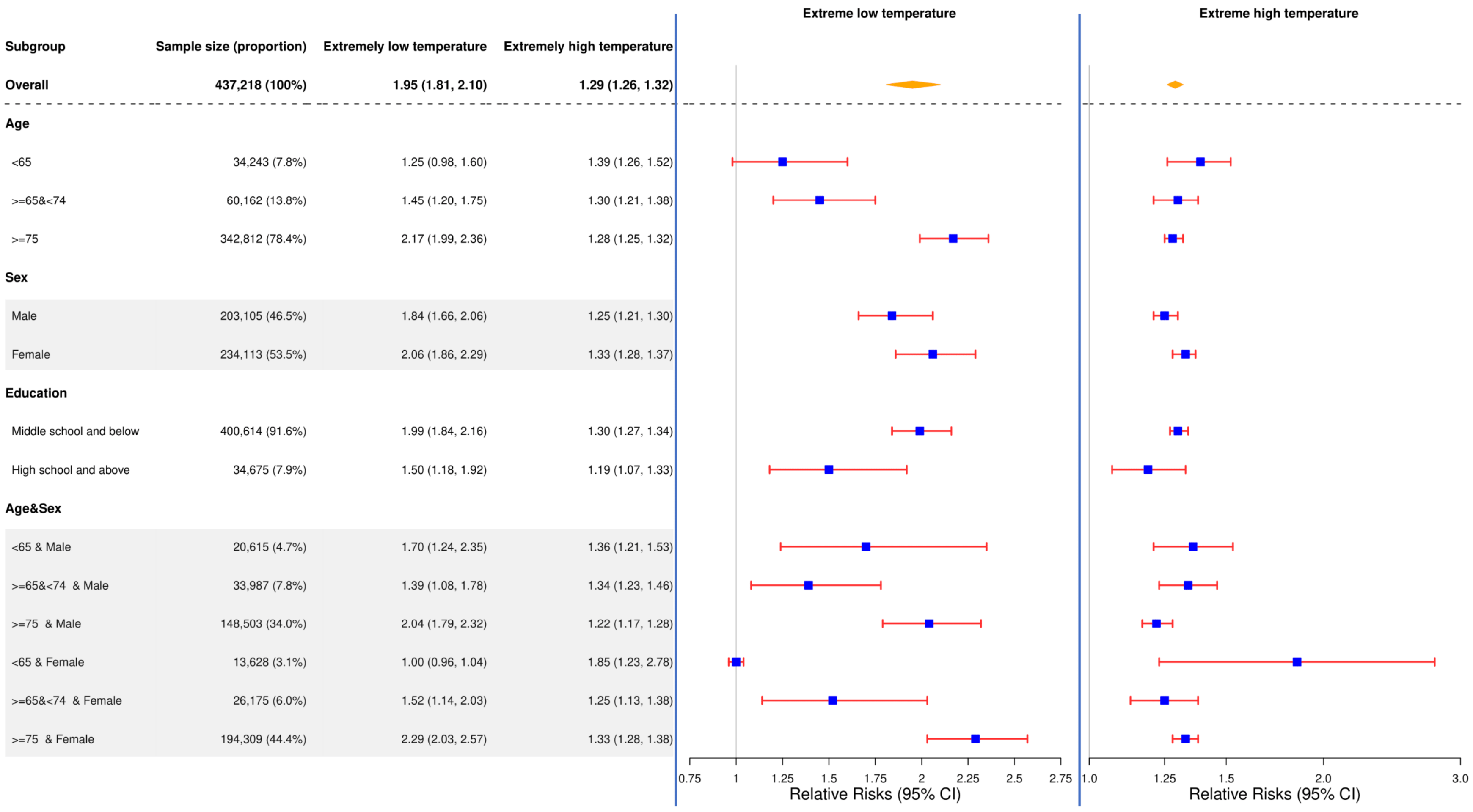

Supplementary Fig. 3. The cumulative relative risks (means and 95% confidence intervals) of overall neurodegenerative diseases death comparing extreme high temperatures (the 97.5th percentile) and extreme low temperatures (the 2.5th percentile) to the minimum-mortality temperatures over lag 0–14 day nationally, stratified by age, sex, education, and combinations of age and sex. Points represent the estimated relative risks. Horizontal lines represent the 95% confidence interval (CI). Source data are provided as a Source Data file.

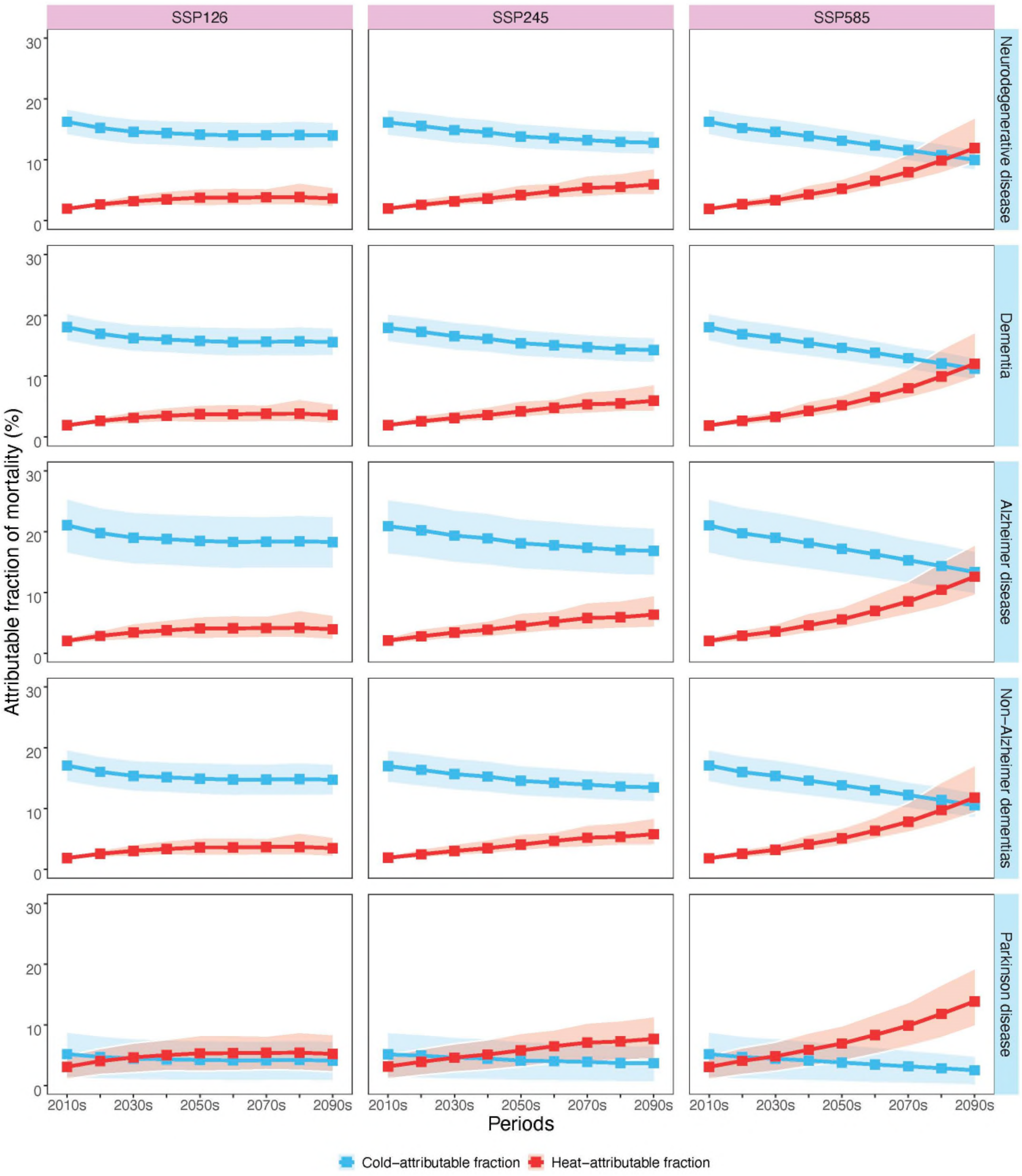

Supplementary Fig. 4. The projected fraction of neurodegenerative disease death associated with non-optimum temperatures in Subtropical monsoon climate zones of China under three climate change scenarios (SSP126, SSP245 and SSP585) for every decade from the 2010s to 2090s. Estimates are reported as GCM-ensemble averages. The points denote the mean estimates and the shaded areas represent their empirical 95% confidence intervals computed from Monte Carlo simulations (1,000 samples). SSP = Shared Socioeconomic Pathway. GCM = General climate models. Source data are provided as a Source Data file.

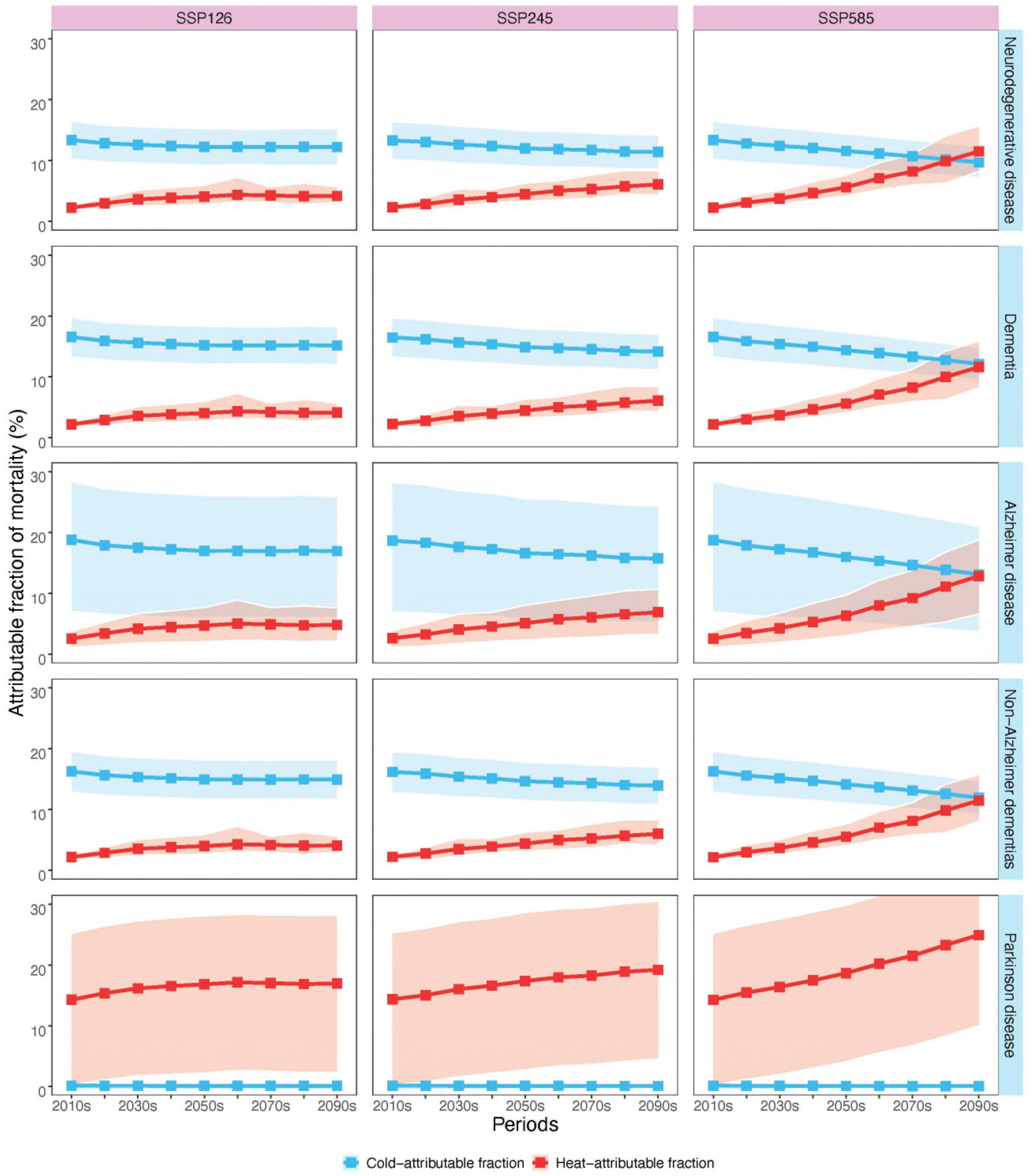

Supplementary Fig. 5. The projected fraction of neurodegenerative disease death associated with non-optimum temperatures in Temperate monsoon climate zones of China under three climate change scenarios (SSP126, SSP245 and SSP585) for every decade from the 2010s to 2090s. Estimates are reported as GCM-ensemble averages. The points denote the mean estimates and the shaded areas represent their empirical 95% confidence intervals computed from Monte Carlo simulations (1,000 samples). SSP = Shared Socioeconomic Pathway. GCM = General climate models. Source data are provided as a Source Data file.

Supplementary Table 4. The average of temperature-attributable number (means and empirical 95% confidence intervals) of neurodegenerative disease death in each decade between 1980 and 2009, classified by climatic zones.

Diseases	Attributable number	Nationwide	Temperate monsoon climate	Subtropical monsoon climate
Neurodegenerative disease	Cold	99560 (83032, 115909)	29603 (22640, 36461)	69957 (60392, 79449)
	Heat	9999 (7511, 13226)	3753 (2830, 4926)	6246 (4682, 8300)
	Total	109559 (93754, 125297)	33356 (26547, 40069)	76203 (67206, 85228)
Dementia	Cold	97973 (83010, 112766)	32091 (25783, 38282)	65883 (57227, 74484)
	Heat	8469 (6292, 11285)	3229 (2407, 4276)	5240 (3885, 7009)
	Total	106442 (92078, 120618)	35320 (29132, 41361)	71122 (62947, 79257)
Alzheimer disease	Cold	21014 (15398, 26191)	2702 (1046, 4073)	18312 (14351, 22118)
	Heat	1612 (1011, 2342)	277 (109, 445)	1335 (901, 1896)
	Total	22625 (17107, 27593)	2979 (1357, 4294)	19646 (15750, 23298)
Non-Alzheimer dementias	Cold	76802 (63201, 90298)	29218 (23106, 35198)	47584 (40095, 55100)
	Heat	6839 (4998, 9184)	2951 (2176, 3932)	3888 (2823, 5252)
	Total	83642 (70377, 96628)	32169 (26154, 38011)	51472 (44223, 58618)
Parkinson disease	Cold	3040 (699, 5203)	20 (-149, 161)	3020 (847, 5041)
	Heat	4126 (195, 7349)	2771 (-131, 5013)	1355 (326, 2336)
	Total	7166 (2573, 10928)	2792 (-14, 4940)	4374 (2587, 5988)

Notes: The empirical 95% confidence intervals computed from Monte Carlo simulations (1,000 samples).

Supplementary Table 5. The differences in heat-attributable fraction (%, means and empirical 95% confidence intervals) of neurodegenerative disease death in 2010-2090 compared with 1980–2009 under three climate change scenarios (SSP126, SSP245 and SSP585), classified by climatic zones and periods.

Diseases	Period	Nationwide			Temperate monsoon climate			Subtropical monsoon climate		
		SSP126	SSP245	SSP585	SSP126	SSP245	SSP585	SSP126	SSP245	SSP585
Neurodegenerative disease	2010s	0.6 (-0.1, 1.1)	0.4 (-0.1, 0.9)	0.4 (-0.1, 0.9)	0.6 (-0.1, 1.1)	0.4 (0.0, 1.0)	0.4 (-0.1, 0.9)	0.6 (-0.1, 1.0)	0.4 (-0.1, 0.8)	0.4 (-0.2, 0.9)
	2030s	1.9 (1.1, 3.0)	1.6 (0.7, 3.0)	1.9 (1.0, 2.9)	1.9 (0.9, 3.2)	1.7 (0.5, 3.4)	1.9 (1.0, 2.9)	1.8 (1.2, 2.7)	1.6 (0.8, 2.6)	1.8 (1.0, 2.8)
	2050s	2.4 (1.0, 4.0)	2.6 (1.5, 4.4)	3.8 (2.5, 5.6)	2.4 (1.1, 4.0)	2.6 (1.4, 4.3)	3.8 (2.5, 5.7)	2.4 (0.9, 3.9)	2.6 (1.6, 4.5)	3.7 (2.4, 5.5)
	2070s	2.5 (1.4, 3.7)	3.6 (2.2, 5.8)	6.4 (4.3, 9.5)	2.6 (1.5, 3.6)	3.4 (2.1, 5.6)	6.4 (3.9, 9.3)	2.5 (1.3, 3.9)	3.8 (2.4, 5.9)	6.5 (4.6, 9.7)
	2090s	2.4 (1.1, 4.0)	4.3 (2.7, 6.6)	10.0 (7.1, 14.8)	2.5 (1.4, 3.9)	4.2 (2.5, 6.4)	9.7 (6.2, 13.9)	2.3 (0.8, 4.2)	4.4 (2.8, 6.9)	10.4 (8, 15.7)
Dementia	2010s	0.6 (-0.1, 1.1)	0.4 (-0.1, 0.9)	0.4 (-0.1, 0.9)	0.6 (-0.2, 1.1)	0.4 (0.0, 1.0)	0.4 (-0.1, 0.9)	0.6 (-0.1, 1.0)	0.4 (-0.1, 0.8)	0.4 (-0.2, 0.9)
	2030s	1.9 (1.1, 3.0)	1.7 (0.7, 3.0)	1.9 (1.0, 2.9)	2.0 (1.0, 3.3)	1.7 (0.5, 3.4)	1.9 (1.0, 3.0)	1.8 (1.2, 2.7)	1.6 (0.8, 2.6)	1.8 (1.0, 2.8)
	2050s	2.4 (1.0, 4.0)	2.6 (1.5, 4.5)	3.8 (2.5, 5.7)	2.4 (1.1, 4.1)	2.6 (1.4, 4.3)	3.8 (2.5, 5.8)	2.4 (0.9, 4.0)	2.7 (1.7, 4.6)	3.7 (2.4, 5.6)
	2070s	2.5 (1.4, 3.8)	3.7 (2.2, 5.9)	6.5 (4.3, 9.6)	2.6 (1.5, 3.6)	3.5 (2.1, 5.7)	6.5 (4.0, 9.4)	2.5 (1.3, 3.9)	3.8 (2.4, 6.0)	6.5 (4.6, 9.9)
	2090s	2.4 (1.1, 4.1)	4.3 (2.7, 6.7)	10.2 (7.2, 15.0)	2.5 (1.4, 3.9)	4.3 (2.5, 6.5)	9.8 (6.3, 14.1)	2.3 (0.8, 4.2)	4.4 (2.8, 7.0)	10.5 (8.1, 16.0)
Alzheimer disease	2010s	0.7 (-0.2, 1.3)	0.5 (-0.2, 1.1)	0.5 (-0.2, 1.1)	0.7 (-0.2, 1.5)	0.5 (-0.1, 1.2)	0.5 (-0.2, 1.2)	0.6 (-0.2, 1.2)	0.4 (-0.2, 0.9)	0.4 (-0.2, 1.0)
	2030s	2.2 (1.1, 3.7)	1.8 (0.8, 3.5)	2.1 (1.0, 3.5)	2.3 (0.9, 4.1)	2.0 (0.6, 4.1)	2.2 (0.9, 3.8)	2.0 (1.2, 3.2)	1.7 (0.9, 2.9)	2.0 (1.1, 3.1)
	2050s	2.8 (1.0, 4.8)	2.9 (1.5, 5.2)	4.1 (2.3, 6.5)	2.8 (1.0, 5.1)	3.0 (1.3, 5.4)	4.3 (2.0, 7.0)	2.7 (0.9, 4.5)	2.9 (1.6, 5.1)	4.0 (2.6, 6.1)
	2070s	2.9 (1.3, 4.7)	4.0 (2.1, 6.8)	7.0 (4.1, 10.7)	3.0 (1.3, 4.8)	3.9 (1.7, 6.9)	7.1 (3.3, 11.1)	2.7 (1.3, 4.5)	4.1 (2.4, 6.7)	6.9 (4.8, 10.4)
	2090s	2.7 (1.0, 5.1)	4.7 (2.5, 7.9)	10.9 (6.7, 16.4)	2.9 (1.2, 5.2)	4.8 (2.2, 8.0)	10.8 (5.4, 16.3)	2.5 (0.8, 4.9)	4.7 (2.9, 7.8)	11 (8.0, 16.5)
Non-Alzheimer dementias	2010s	0.6 (-0.1, 1.1)	0.4 (-0.1, 0.9)	0.4 (-0.1, 0.9)	0.6 (-0.1, 1.1)	0.4 (0.0, 1.0)	0.4 (-0.1, 0.9)	0.6 (-0.1, 1.0)	0.4 (-0.1, 0.8)	0.4 (-0.1, 0.9)
	2030s	1.8 (1.0, 2.9)	1.6 (0.6, 3.0)	1.8 (1.0, 2.9)	1.9 (0.9, 3.2)	1.7 (0.5, 3.4)	1.9 (1.0, 3.0)	1.7 (1.1, 2.6)	1.5 (0.8, 2.6)	1.8 (1.0, 2.8)
	2050s	2.3 (1.0, 4.0)	2.6 (1.5, 4.4)	3.7 (2.4, 5.7)	2.4 (1.1, 4.0)	2.6 (1.4, 4.3)	3.8 (2.5, 5.8)	2.3 (0.9, 3.9)	2.6 (1.6, 4.5)	3.7 (2.3, 5.5)
	2070s	2.5 (1.3, 3.7)	3.6 (2.2, 5.8)	6.4 (4.2, 9.6)	2.6 (1.5, 3.6)	3.4 (2.0, 5.6)	6.4 (3.9, 9.3)	2.4 (1.2, 3.7)	3.7 (2.3, 5.9)	6.4 (4.5, 9.8)
	2090s	2.3 (1.1, 4.0)	4.3 (2.6, 6.6)	10.0 (7.0, 15.0)	2.5 (1.4, 3.9)	4.2 (2.5, 6.4)	9.7 (6.2, 14.0)	2.2 (0.8, 4.1)	4.3 (2.7, 6.8)	10.4 (7.8, 15.9)
Parkinson disease	2010s	1.0 (0.1, 1.6)	0.7 (0.1, 1.4)	0.7 (0.0, 1.5)	1.1 (0.2, 1.8)	0.9 (0.3, 1.7)	0.9 (0.1, 1.6)	0.8 (-0.1, 1.4)	0.5 (-0.2, 1.1)	0.5 (-0.2, 1.3)
	2030s	2.7 (1.3, 4.2)	2.3 (1.1, 4.0)	2.6 (1.4, 4.0)	3.0 (1.2, 4.6)	2.6 (1.0, 4.7)	3.0 (1.6, 4.3)	2.4 (1.4, 3.7)	2.0 (1.1, 3.3)	2.3 (1.3, 3.6)
	2050s	3.3 (1.5, 5.5)	3.5 (2.0, 5.9)	4.8 (3.1, 6.9)	3.7 (1.9, 6.0)	3.9 (2.2, 6.2)	5.3 (3.4, 7.2)	3.0 (1.2, 5.1)	3.2 (1.8, 5.5)	4.4 (2.7, 6.6)
	2070s	3.5 (1.9, 5.3)	4.6 (2.8, 7.3)	7.7 (5.0, 11.2)	3.9 (2.3, 5.7)	4.8 (2.9, 7.3)	8.1 (5.0, 11.4)	3.1 (1.5, 5.0)	4.5 (2.6, 7.2)	7.3 (5.0, 11.0)
	2090s	3.4 (1.6, 5.8)	5.4 (3.4, 8.2)	11.4 (7.9, 16.6)	3.8 (2.2, 6.0)	5.7 (3.6, 8.4)	11.5 (7.6, 16.1)	2.9 (1.0, 5.5)	5.1 (3.2, 8.1)	11.3 (8.1, 17.0)

Notes: The empirical 95% confidence intervals computed from Monte Carlo simulations (1,000 samples). SSP = Shared Socioeconomic Pathway.

Supplementary Table 6. The differences in cold-attributable fraction (%, means and empirical 95% confidence intervals) of neurodegenerative disease death in 2010-2090 compared with 1980–2009 under three climate change scenarios (SSP126, SSP245 and SSP585), classified by climatic zones and periods.

Diseases	Period	Nationwide			Temperate monsoon climate			Subtropical monsoon climate		
		SSP126	SSP245	SSP585	SSP126	SSP245	SSP585	SSP126	SSP245	SSP585
Neurodegenerative disease	2010s	-0.7 (-1.4, -0.2)	-0.7 (-1.4, -0.2)	-0.6 (-1.4, -0.1)	-0.6 (-1.0, -0.2)	-0.5 (-1.0, -0.1)	-0.5 (-1.0, 0.0)	-0.9 (-1.8, -0.2)	-0.8 (-1.9, -0.2)	-0.7 (-1.8, -0.2)
	2030s	-2.0 (-2.8, -1.2)	-1.7 (-2.3, -1.0)	-1.9 (-2.7, -1.2)	-1.4 (-2.1, -0.6)	-1.2 (-1.8, -0.7)	-1.5 (-2.0, -0.9)	-2.5 (-3.6, -1.8)	-2.1 (-2.8, -1.2)	-2.4 (-3.3, -1.5)
	2050s	-2.4 (-3.4, -1.5)	-2.5 (-3.6, -1.5)	-3.1 (-4.1, -2.2)	-1.7 (-2.6, -1.1)	-1.9 (-2.8, -1.1)	-2.3 (-3.1, -1.6)	-3.0 (-4.2, -2.0)	-3.1 (-4.3, -2.0)	-3.9 (-5.0, -2.8)
	2070s	-2.4 (-3.3, -1.6)	-2.9 (-4.0, -2.3)	-4.3 (-5.6, -3.3)	-1.7 (-2.6, -1.1)	-2.1 (-2.9, -1.5)	-3.2 (-4.3, -2.1)	-3.1 (-4.1, -2.0)	-3.7 (-5.0, -3.0)	-5.4 (-6.8, -4.4)
	2090s	-2.4 (-3.6, -1.5)	-3.3 (-4.4, -2.6)	-5.6 (-7.1, -4.5)	-1.7 (-2.7, -1.0)	-2.4 (-3.4, -1.7)	-4.2 (-5.5, -3.2)	-3.1 (-4.5, -2.0)	-4.2 (-5.4, -3.4)	-7.0 (-8.7, -5.9)
Dementia	2010s	-0.8 (-1.5, -0.2)	-0.8 (-1.6, -0.2)	-0.7 (-1.6, -0.1)	-0.7 (-1.2, -0.3)	-0.7 (-1.1, -0.2)	-0.6 (-1.2, 0.0)	-1.0 (-1.9, -0.2)	-0.9 (-2.0, -0.2)	-0.8 (-2.0, -0.2)
	2030s	-2.2 (-3.2, -1.3)	-1.9 (-2.6, -1.1)	-2.2 (-3.0, -1.3)	-1.7 (-2.5, -0.7)	-1.5 (-2.2, -0.8)	-1.8 (-2.4, -1.0)	-2.7 (-3.9, -1.9)	-2.3 (-3.0, -1.3)	-2.6 (-3.6, -1.6)
	2050s	-2.7 (-3.9, -1.7)	-2.8 (-4.0, -1.8)	-3.5 (-4.6, -2.5)	-2.1 (-3.2, -1.3)	-2.2 (-3.4, -1.4)	-2.8 (-3.7, -2.1)	-3.2 (-4.6, -2.2)	-3.4 (-4.7, -2.2)	-4.2 (-5.4, -3.0)
	2070s	-2.7 (-3.7, -1.8)	-3.3 (-4.5, -2.6)	-4.9 (-6.3, -3.7)	-2.1 (-3.1, -1.4)	-2.6 (-3.5, -1.9)	-3.8 (-5.2, -2.6)	-3.4 (-4.4, -2.2)	-4.1 (-5.4, -3.3)	-5.9 (-7.4, -4.8)
	2090s	-2.8 (-4.0, -1.7)	-3.7 (-4.9, -2.9)	-6.3 (-8.0, -5.2)	-2.1 (-3.2, -1.3)	-3.0 (-4.0, -2.2)	-5.1 (-6.5, -4.0)	-3.4 (-4.9, -2.2)	-4.5 (-5.8, -3.7)	-7.6 (-9.5, -6.4)
Alzheimer disease	2010s	-1.1 (-2.0, -0.3)	-1.0 (-2.0, -0.2)	-0.9 (-2.0, 0.0)	-1.0 (-1.8, -0.3)	-0.9 (-1.8, -0.2)	-0.8 (-1.8, 0.2)	-1.2 (-2.2, -0.3)	-1.1 (-2.3, -0.3)	-0.9 (-2.3, -0.2)
	2030s	-2.8 (-4.2, -1.5)	-2.3 (-3.3, -1.2)	-2.6 (-3.9, -1.4)	-2.3 (-3.7, -0.9)	-1.9 (-3.0, -0.9)	-2.3 (-3.5, -1.1)	-3.2 (-4.6, -2.0)	-2.6 (-3.6, -1.5)	-3.0 (-4.3, -1.8)
	2050s	-3.3 (-5.0, -1.9)	-3.4 (-5.1, -1.9)	-4.2 (-5.8, -2.7)	-2.8 (-4.6, -1.3)	-2.9 (-4.7, -1.4)	-3.6 (-5.3, -1.9)	-3.8 (-5.4, -2.4)	-3.9 (-5.4, -2.4)	-4.8 (-6.3, -3.4)
	2070s	-3.4 (-4.8, -1.9)	-3.9 (-5.6, -2.6)	-5.8 (-7.9, -3.9)	-2.9 (-4.4, -1.4)	-3.3 (-4.9, -1.7)	-4.9 (-7.3, -2.5)	-3.9 (-5.2, -2.4)	-4.6 (-6.2, -3.5)	-6.7 (-8.6, -5.2)
	2090s	-3.4 (-5.3, -1.8)	-4.5 (-6.3, -3.0)	-7.5 (-10.0, 5.2)	-2.9 (-4.8, -1.2)	-3.8 (-5.7, -2.0)	-6.5 (-9.2, -3.5)	-3.9 (-5.9, -2.5)	-5.1 (-6.8, -4.0)	-8.6 (-10.9, -6.8)
Non-Alzheimer dementias	2010s	-0.8 (-1.5, -0.2)	-0.7 (-1.5, -0.2)	-0.7 (-1.5, -0.1)	-0.7 (-1.2, -0.3)	-0.6 (-1.1, -0.2)	-0.6 (-1.2, 0.0)	-0.9 (-1.8, -0.2)	-0.8 (-1.9, -0.2)	-0.8 (-1.9, -0.2)
	2030s	-2.1 (-3.0, -1.3)	-1.8 (-2.5, -1.0)	-2.1 (-2.9, -1.3)	-1.6 (-2.4, -0.7)	-1.4 (-2.2, -0.8)	-1.7 (-2.3, -1.0)	-2.6 (-3.7, -1.8)	-2.2 (-2.9, -1.3)	-2.5 (-3.5, -1.5)
	2050s	-2.5 (-3.7, -1.7)	-2.7 (-3.9, -1.7)	-3.4 (-4.4, -2.4)	-2.0 (-3.1, -1.3)	-2.2 (-3.3, -1.3)	-2.7 (-3.7, -2.0)	-3.1 (-4.4, -2.0)	-3.3 (-4.5, -2.0)	-4.0 (-5.2, -2.8)
	2070s	-2.6 (-3.6, -1.7)	-3.2 (-4.3, -2.5)	-4.7 (-6.1, -3.6)	-2.1 (-3.0, -1.3)	-2.5 (-3.4, -1.8)	-3.8 (-5.1, -2.6)	-3.2 (-4.3, -2.1)	-3.9 (-5.2, -3.1)	-5.6 (-7.1, -4.6)
	2090s	-2.6 (-3.9, -1.6)	-3.6 (-4.8, -2.8)	-6.1 (-7.8, -5.0)	-2.0 (-3.2, -1.2)	-2.9 (-3.9, -2.1)	-4.9 (-6.4, -3.8)	-3.2 (-4.7, -2)	-4.3 (-5.7, -3.5)	-7.3 (-9.2, -6.1)
Parkinson disease	2010s	-0.2 (-0.7, 0.1)	-0.2 (-0.7, 0.0)	-0.2 (-0.7, 0.1)	0.0 (-0.2, 0.2)	0.0 (-0.2, 0.2)	0.0 (-0.2, 0.2)	-0.5 (-1.1, 0.1)	-0.5 (-1.2, -0.1)	-0.4 (-1.1, 0.0)
	2030s	-0.6 (-1.2, -0.1)	-0.5 (-1.0, -0.1)	-0.6 (-1.1, -0.1)	0.0 (-0.3, 0.3)	0.0 (-0.3, 0.3)	0.0 (-0.4, 0.3)	-1.2 (-2.1, -0.5)	-1.0 (-1.6, -0.4)	-1.1 (-1.9, -0.5)
	2050s	-0.7 (-1.4, -0.2)	-0.8 (-1.4, -0.1)	-0.9 (-1.6, -0.2)	0.0 (-0.4, 0.3)	-0.1 (-0.4, 0.4)	-0.1 (-0.5, 0.4)	-1.4 (-2.4, -0.6)	-1.5 (-2.5, -0.6)	-1.8 (-2.8, -0.8)
	2070s	-0.8 (-1.4, -0.2)	-0.9 (-1.5, -0.2)	-1.2 (-2.2, -0.3)	0.0 (-0.4, 0.3)	-0.1 (-0.4, 0.4)	-0.1 (-0.6, 0.5)	-1.5 (-2.4, -0.6)	-1.7 (-2.7, -0.8)	-2.4 (-3.8, -1.0)
	2090s	-0.8 (-1.5, -0.2)	-1.0 (-1.7, -0.2)	-1.6 (-2.7, -0.3)	0.0 (-0.4, 0.3)	-0.1 (-0.5, 0.4)	-0.1 (-0.7, 0.6)	-1.5 (-2.5, -0.6)	-1.9 (-3.0, -0.8)	-3.0 (-4.7, -1.3)

Notes: The empirical 95% confidence intervals computed from Monte Carlo simulations (1,000 samples). SSP = Shared Socioeconomic Pathway.

Supplementary Table 7. The differences in heat-attributable number (means and empirical 95% confidence intervals) of neurodegenerative disease death in 2010-2090 compared with 1980–2009 under three climate change scenarios (SSP126, SSP245 and SSP585), classified by climatic zones and periods.

Diseases	Period	Nationwide			Temperate monsoon climate			Subtropical monsoon climate		
		SSP126	SSP245	SSP585	SSP126	SSP245	SSP585	SSP126	SSP245	SSP585
Neurodegenerative disease	2010s	3244 (208, 6166)	3089 (1112, 5361)	3076 (781, 5942)	1051 (26, 1966)	997 (343, 1730)	979 (223, 1910)	2193 (182, 4201)	2092 (769, 3630)	2096 (558, 4031)
	2030s	9412 (5060, 12908)	8670 (5407, 13098)	12152 (7951, 18192)	2885 (1497, 4020)	2673 (1652, 4001)	3703 (2458, 5424)	6528 (3562, 8888)	5997 (3754, 9096)	8450 (5493, 12768)
	2050s	13129 (6883, 18818)	15997 (9294, 24666)	25059 (14114, 41869)	3984 (2126, 5595)	4791 (2824, 7232)	7445 (4248, 12312)	9145 (4757, 13223)	11207 (6470, 17434)	17613 (9866, 29557)
	2070s	14941 (8372, 20545)	22700 (12964, 35901)	44140 (23203, 73875)	4512 (2576, 6284)	6767 (3943, 10420)	12785 (6799, 20920)	10429 (5797, 14261)	15933 (9021, 25481)	31355 (16403, 52955)
	2090s	15384 (6455, 26185)	26371 (13744, 39683)	69946 (31899, 114617)	4637 (1986, 7756)	7785 (4172, 11556)	20018 (9149, 32119)	10747 (4469, 18429)	18586 (9572, 28126)	49927 (22750, 82498)
Dementia	2010s	2788 (177, 5322)	2653 (943, 4621)	2643 (664, 5127)	923 (22, 1737)	877 (298, 1528)	860 (197, 1685)	1865 (155, 3585)	1777 (645, 3093)	1783 (466, 3441)
	2030s	8109 (4342, 11147)	7475 (4637, 11332)	10482 (6838, 15703)	2545 (1311, 3558)	2358 (1450, 3541)	3272 (2165, 4802)	5564 (3030, 7589)	5117 (3187, 7790)	7211 (4673, 10901)
	2050s	11328 (5911, 16281)	13811 (7998, 21377)	21679 (12172, 36351)	3522 (1866, 4953)	4236 (2487, 6412)	6607 (3757, 10960)	7806 (4045, 11328)	9575 (5511, 14965)	15073 (8415, 25391)
	2070s	12898 (7218, 17826)	19625 (11169, 31139)	38275 (20028, 64176)	3993 (2277, 5588)	6000 (3488, 9260)	11367 (6011, 18618)	8905 (4941, 12239)	13624 (7681, 21879)	26908 (14017, 45557)
	2090s	13271 (5548, 22700)	22814 (11841, 34464)	60703 (27587, 99613)	4104 (1752, 6908)	6903 (3689, 10281)	17826 (8113, 28638)	9167 (3797, 15791)	15911 (8152, 24183)	42877 (19474, 70975)
Alzheimer disease	2010s	549 (15, 1080)	523 (166, 939)	521 (119, 1037)	81 (6, 166)	78 (27, 146)	77 (17, 156)	468 (9, 914)	444 (139, 793)	444 (102, 880)
	2030s	1629 (809, 2333)	1491 (851, 2330)	2112 (1300, 3306)	222 (91, 351)	204 (90, 336)	285 (136, 460)	1407 (718, 1982)	1287 (761, 1994)	1827 (1165, 2847)
	2050s	2279 (1110, 3420)	2788 (1531, 4456)	4385 (2332, 7551)	303 (124, 476)	363 (164, 596)	567 (262, 1012)	1976 (985, 2945)	2425 (1367, 3859)	3817 (2071, 6539)
	2070s	2606 (1371, 3755)	3970 (2159, 6505)	7730 (3879, 13210)	344 (158, 549)	513 (236, 864)	964 (425, 1672)	2262 (1213, 3206)	3457 (1923, 5640)	6767 (3454, 11538)
	2090s	2691 (1057, 4760)	4629 (2284, 7205)	12228 (5395, 20323)	354 (126, 661)	588 (261, 958)	1490 (606, 2505)	2337 (931, 4099)	4041 (2022, 6247)	10738 (4789, 17817)
Non-Alzheimer dementias	2010s	2232 (154, 4296)	2124 (756, 3729)	2116 (531, 4142)	841 (14, 1592)	797 (263, 1401)	783 (176, 1548)	1391 (139, 2704)	1327 (493, 2329)	1333 (355, 2594)
	2030s	6463 (3414, 8952)	5967 (3673, 9101)	8348 (5415, 12536)	2319 (1182, 3258)	2150 (1320, 3238)	2982 (1973, 4384)	4143 (2231, 5694)	3816 (2353, 5863)	5366 (3442, 8151)
	2050s	9025 (4683, 13023)	10993 (6331, 17099)	17249 (9639, 29069)	3214 (1689, 4537)	3866 (2265, 5871)	6028 (3416, 10023)	5811 (2994, 8486)	7127 (4066, 11228)	11221 (6223, 19046)
	2070s	10264 (5717, 14324)	15614 (8823, 24880)	30465 (15846, 51365)	3642 (2060, 5119)	5477 (3173, 8468)	10382 (5468, 17056)	6621 (3657, 9205)	10137 (5650, 16412)	20083 (10378, 34309)
	2090s	10553 (4397, 18096)	18138 (9370, 27541)	48354 (21831, 79793)	3744 (1587, 6298)	6303 (3356, 9416)	16300 (7385, 26259)	6809 (2810, 11798)	11835 (6014, 18125)	32055 (14447, 53534)
Parkinson disease	2010s	554 (70, 1088)	533 (202, 971)	531 (158, 1049)	180 (34, 342)	174 (67, 313)	172 (57, 324)	374 (35, 746)	359 (135, 658)	358 (100, 725)
	2030s	1511 (742, 2206)	1384 (800, 2184)	1886 (1172, 2983)	456 (231, 669)	420 (247, 652)	548 (346, 852)	1055 (511, 1537)	964 (552, 1531)	1339 (826, 2131)
	2050s	2034 (1021, 3052)	2452 (1397, 3855)	3663 (2067, 6187)	591 (306, 881)	703 (419, 1088)	996 (595, 1644)	1443 (715, 2171)	1749 (977, 2767)	2667 (1472, 4543)
	2070s	2297 (1252, 3273)	3362 (1882, 5460)	6160 (3315, 10446)	660 (374, 933)	928 (532, 1484)	1594 (911, 2641)	1637 (878, 2340)	2434 (1349, 3976)	4567 (2404, 7806)
	2090s	2355 (977, 3887)	3851 (2008, 5882)	9389 (4450, 15514)	666 (295, 1020)	1048 (574, 1571)	2325 (1182, 3733)	1689 (682, 2867)	2803 (1434, 4311)	7064 (3268, 11782)

Notes: The empirical 95% confidence intervals computed from Monte Carlo simulations (1,000 samples). SSP = Shared Socioeconomic Pathway.

Supplementary Table 8. The differences in cold-attributable number (means and empirical 95% confidence intervals) of neurodegenerative disease death in 2010-2090 compared with 1980–2009 under three climate change scenarios (SSP126, SSP245 and SSP585), classified by climatic zones and periods.

Diseases	Period	Nationwide			Temperate monsoon climate			Subtropical monsoon climate		
		SSP126	SSP245	SSP585	SSP126	SSP245	SSP585	SSP126	SSP245	SSP585
Neurodegenerative disease	2010s	-5126 (-8866, -1687)	-5019 (-8618, -2389)	-5063 (-7939, -2788)	-1078 (-1967, -366)	-1058 (-1887, -509)	-1057 (-1764, -582)	-4048 (-6899, -1320)	-3961 (-6730, -1880)	-4006 (-6175, -2206)
	2030s	-11602 (-17371, -7157)	-11114 (-16792, -8056)	-13138 (-20204, -8296)	-2513 (-3803, -1524)	-2366 (-3668, -1609)	-2827 (-4507, -1771)	-9089 (-13568, -5633)	-8748 (-13124, -6448)	-10311 (-15698, -6525)
	2050s	-14585 (-20948, -10572)	-16825 (-26131, -13389)	-21498 (-34196, -16409)	-3129 (-4675, -2118)	-3631 (-5748, -2646)	-4675 (-7648, -3297)	-11457 (-16273, -8453)	-13194 (-20383, -10743)	-16823 (-26547, -13111)
	2070s	-15926 (-22508, -11593)	-20815 (-32990, -13859)	-31392 (-50418, -22876)	-3449 (-4965, -2358)	-4501 (-7374, -2903)	-6904 (-11573, -4763)	-12477 (-17543, -9235)	-16313 (-25615, -10956)	-24488 (-38844, -18112)
	2090s	-15734 (-21428, -9749)	-22569 (-33774, -15517)	-41285 (-62950, -28147)	-3391 (-4847, -1975)	-4921 (-7511, -3205)	-9216 (-14760, -5972)	-12343 (-16580, -7774)	-17648 (-26263, -12312)	-32069 (-48190, -22175)
Dementia	2010s	-4855 (-8401, -1690)	-4748 (-8126, -2300)	-4789 (-7542, -2677)	-1141 (-2051, -423)	-1119 (-1961, -558)	-1119 (-1847, -636)	-3714 (-6349, -1267)	-3629 (-6165, -1742)	-3670 (-5695, -2041)
	2030s	-11019 (-16372, -6882)	-10513 (-15828, -7671)	-12489 (-19184, -8056)	-2665 (-3957, -1657)	-2500 (-3822, -1749)	-3004 (-4734, -1951)	-8354 (-12415, -5226)	-8013 (-12006, -5922)	-9485 (-14449, -6105)
	2050s	-13831 (-19883, -10081)	-15972 (-24698, -12785)	-20451 (-32457, -15661)	-3312 (-4905, -2292)	-3848 (-6012, -2900)	-4962 (-8027, -3608)	-10519 (-14978, -7790)	-12124 (-18686, -9884)	-15489 (-24430, -12054)
	2070s	-15115 (-21291, -11013)	-19777 (-31297, -13194)	-29918 (-48010, -21886)	-3652 (-5194, -2550)	-4773 (-7738, -3117)	-7331 (-12155, -5179)	-11463 (-16097, -8463)	-15004 (-23559, -10077)	-22586 (-35855, -16706)
	2090s	-14934 (-20274, -9220)	-21466 (-31993, -14768)	-39438 (-60126, -26892)	-3594 (-5050, -2112)	-5219 (-7870, -3451)	-9792 (-15523, -6430)	-11340 (-15223, -7108)	-16247 (-24124, -11317)	-29646 (-44603, -20461)
Alzheimer disease	2010s	-1088 (-1944, -325)	-1060 (-1863, -489)	-1071 (-1744, -567)	-116 (-214, -32)	-116 (-202, -52)	-115 (-192, -51)	-972 (-1730, -293)	-944 (-1661, -437)	-956 (-1552, -517)
	2030s	-2487 (-3776, -1456)	-2374 (-3649, -1658)	-2815 (-4403, -1716)	-263 (-428, -123)	-253 (-406, -136)	-300 (-499, -143)	-2224 (-3348, -1332)	-2121 (-3244, -1522)	-2515 (-3904, -1573)
	2050s	-3134 (-4574, -2170)	-3600 (-5690, -2713)	-4613 (-7483, -3350)	-329 (-516, -171)	-385 (-650, -207)	-490 (-847, -262)	-2805 (-4059, -1999)	-3215 (-5041, -2507)	-4124 (-6635, -3088)
	2070s	-3423 (-4915, -2396)	-4466 (-7212, -2893)	-6752 (-11087, -4737)	-359 (-549, -191)	-476 (-825, -247)	-722 (-1277, -381)	-3064 (-4366, -2205)	-3990 (-6387, -2646)	-6031 (-9810, -4355)
	2090s	-3389 (-4716, -2040)	-4836 (-7349, -3235)	-8894 (-13890, -5840)	-354 (-552, -171)	-514 (-832, -268)	-956 (-1624, -494)	-3035 (-4164, -1869)	-4322 (-6516, -2967)	-7938 (-12266, -5346)
Non-Alzheimer dementias	2010s	-3753 (-6528, -1321)	-3675 (-6319, -1771)	-3705 (-5869, -2059)	-1023 (-1860, -378)	-1002 (-1774, -492)	-1002 (-1677, -566)	-2731 (-4668, -943)	-2674 (-4545, -1279)	-2702 (-4192, -1493)
	2030s	-8506 (-12722, -5289)	-8113 (-12293, -5825)	-9644 (-14936, -6170)	-2396 (-3574, -1479)	-2243 (-3450, -1550)	-2699 (-4281, -1744)	-6110 (-9148, -3810)	-5870 (-8842, -4276)	-6946 (-10656, -4426)
	2050s	-10664 (-15473, -7663)	-12334 (-19190, -9668)	-15790 (-25232, -11921)	-2976 (-4436, -2030)	-3457 (-5418, -2569)	-4462 (-7252, -3202)	-7689 (-11036, -5632)	-8877 (-13772, -7099)	-11328 (-17980, -8718)
	2070s	-11656 (-16533, -8401)	-15265 (-24329, -10085)	-23100 (-37342, -16726)	-3284 (-4689, -2265)	-4288 (-6986, -2778)	-6596 (-10990, -4608)	-8372 (-11844, -6136)	-10976 (-17343, -7307)	-16504 (-26352, -12117)
	2090s	-11511 (-15840, -7033)	-16579 (-24883, -11277)	-30461 (-46791, -20602)	-3232 (-4575, -1879)	-4694 (-7109, -3075)	-8816 (-14045, -5749)	-8279 (-11265, -5153)	-11885 (-17773, -8202)	-21645 (-32746, -14852)
Parkinson disease	2010s	-271 (-542, 7)	-268 (-527, -72)	-274 (-534, -75)	-3 (-26, 24)	-3 (-24, 22)	-3 (-28, 24)	-268 (-516, -17)	-266 (-502, -94)	-271 (-506, -100)
	2030s	-583 (-1088, -192)	-589 (-1036, -228)	-656 (-1171, -204)	-5 (-43, 39)	-6 (-45, 41)	-6 (-47, 43)	-578 (-1045, -230)	-583 (-991, -269)	-651 (-1124, -247)
	2050s	-740 (-1244, -270)	-845 (-1542, -332)	-1053 (-1929, -403)	-7 (-52, 48)	-8 (-58, 54)	-9 (-69, 64)	-734 (-1192, -319)	-837 (-1484, -385)	-1044 (-1860, -467)
	2070s	-802 (-1383, -301)	-1030 (-1864, -389)	-1489 (-2672, -571)	-7 (-58, 53)	-9 (-68, 63)	-12 (-88, 82)	-795 (-1325, -354)	-1021 (-1797, -452)	-1477 (-2584, -653)
	2090s	-794 (-1398, -259)	-1102 (-1954, -421)	-1881 (-3231, -699)	-7 (-60, 52)	-9 (-71, 66)	-14 (-105, 97)	-786 (-1338, -311)	-1093 (-1883, -487)	-1866 (-3126, -796)

Notes: The empirical 95% confidence intervals computed from Monte Carlo simulations (1,000 samples). SSP = Shared Socioeconomic Pathway.

Supplementary Table 9. The differences in net change of temperature-attributable fraction (%, means and empirical 95% confidence intervals) of neurodegenerative disease death in 2010-2090 compared with 1980–2009 under three climate change scenarios (SSP126, SSP245 and SSP585), classified by climatic zones and periods.

Diseases	Period	Nationwide			Temperate monsoon climate			Subtropical monsoon climate		
		SSP126	SSP245	SSP585	SSP126	SSP245	SSP585	SSP126	SSP245	SSP585
Neurodegenerative disease	2010s	-0.2 (-0.8, 0.4)	-0.3 (-1, 0.3)	-0.2 (-0.9, 0.3)	0.0 (-0.6, 0.5)	-0.1 (-0.7, 0.3)	-0.1 (-0.7, 0.3)	-0.3 (-1.1, 0.2)	-0.4 (-1.2, 0.2)	-0.3 (-1.1, 0.2)
	2030s	-0.1 (-0.8, 0.8)	0.0 (-0.7, 0.9)	-0.1 (-0.8, 0.7)	0.6 (-0.1, 1.4)	0.5 (-0.3, 1.7)	0.4 (-0.2, 1.4)	-0.7 (-1.5, 0.2)	-0.5 (-1.0, 0.0)	-0.6 (-1.4, 0.1)
	2050s	0.0 (-1.0, 1.3)	0.1 (-0.8, 1.2)	0.7 (-0.5, 2.4)	0.7 (-0.3, 1.7)	0.8 (-0.3, 1.9)	1.5 (0.2, 3.4)	-0.6 (-1.8, 0.8)	-0.5 (-1.3, 0.5)	-0.1 (-1.2, 1.4)
	2070s	0.1 (-1.1, 1.1)	0.7 (-0.7, 2.3)	2.1 (0.2, 4.6)	0.8 (-0.3, 1.7)	1.3 (-0.1, 3.0)	3.2 (1.0, 5.6)	-0.6 (-1.9, 0.4)	0.1 (-1.3, 1.6)	1.1 (-0.5, 3.5)
	2090s	-0.1 (-1.3, 1)	1.0 (-0.6, 2.9)	4.5 (1.7, 8.2)	0.8 (-0.3, 1.8)	1.8 (0.1, 3.6)	5.5 (2.1, 9.1)	-0.9 (-2.3, 0.3)	0.2 (-1.3, 2.1)	3.4 (1.3, 7.3)
Dementia	2010s	-0.3 (-1.0, 0.3)	-0.4 (-1.1, 0.2)	-0.3 (-1.0, 0.2)	-0.1 (-0.7, 0.4)	-0.2 (-0.9, 0.3)	-0.2 (-0.8, 0.2)	-0.4 (-1.2, 0.1)	-0.5 (-1.4, 0.2)	-0.4 (-1.2, 0.2)
	2030s	-0.3 (-1.1, 0.6)	-0.2 (-0.8, 0.6)	-0.3 (-1.1, 0.5)	0.3 (-0.4, 1.1)	0.3 (-0.5, 1.4)	0.1 (-0.6, 1.1)	-0.9 (-1.8, 0.0)	-0.7 (-1.2, -0.2)	-0.8 (-1.7, -0.1)
	2050s	-0.3 (-1.3, 1.0)	-0.2 (-1.1, 0.8)	0.3 (-0.9, 2.0)	0.3 (-0.6, 1.4)	0.4 (-0.7, 1.5)	1.0 (-0.2, 3.0)	-0.8 (-2.1, 0.6)	-0.8 (-1.6, 0.2)	-0.4 (-1.5, 1.0)
	2070s	-0.2 (-1.5, 0.8)	0.3 (-1.1, 1.9)	1.6 (-0.3, 4.1)	0.5 (-0.8, 1.4)	0.9 (-0.6, 2.6)	2.6 (0.4, 5.0)	-0.9 (-2.2, 0.2)	-0.2 (-1.7, 1.3)	0.7 (-1.0, 3.1)
	2090s	-0.4 (-1.7, 0.7)	0.6 (-1.0, 2.4)	3.8 (1.1, 7.6)	0.4 (-0.7, 1.4)	1.3 (-0.3, 3.1)	4.8 (1.4, 8.4)	-1.1 (-2.6, 0.0)	-0.1 (-1.7, 1.7)	2.9 (0.8, 6.8)
Alzheimer disease	2010s	-0.4 (-1.4, 0.4)	-0.5 (-1.5, 0.3)	-0.4 (-1.3, 0.4)	-0.3 (-1.3, 0.5)	-0.4 (-1.4, 0.4)	-0.3 (-1.3, 0.5)	-0.5 (-1.5, 0.3)	-0.6 (-1.6, 0.2)	-0.5 (-1.4, 0.3)
	2030s	-0.6 (-2.0, 0.9)	-0.4 (-1.4, 0.8)	-0.5 (-1.9, 0.8)	0.0 (-1.6, 1.7)	0.0 (-1.2, 1.8)	-0.1 (-1.7, 1.6)	-1.2 (-2.3, 0.1)	-0.9 (-1.6, -0.2)	-1.0 (-2.2, 0.0)
	2050s	-0.5 (-2.2, 1.4)	-0.5 (-2.0, 1.2)	-0.1 (-2.1, 2.4)	0.0 (-1.9, 2.2)	0.1 (-1.9, 2.2)	0.7 (-1.9, 3.7)	-1.1 (-2.6, 0.7)	-1.0 (-2.2, 0.2)	-0.8 (-2.2, 1)
	2070s	-0.5 (-2.3, 1.3)	0.1 (-2.1, 2.6)	1.2 (-1.8, 4.9)	0.2 (-1.9, 2.3)	0.6 (-1.9, 3.5)	2.2 (-1.7, 6.3)	-1.1 (-2.7, 0.4)	-0.5 (-2.3, 1.6)	0.3 (-1.8, 3.4)
	2090s	-0.7 (-2.6, 1.2)	0.3 (-2.0, 3.0)	3.4 (-0.8, 8.2)	0.1 (-2.2, 2.3)	1.0 (-1.8, 4.1)	4.3 (-1.1, 9.9)	-1.4 (-3.0, 0.2)	-0.4 (-2.3, 1.9)	2.4 (-0.4, 6.6)
Non-Alzheimer dementias	2010s	-0.2 (-0.9, 0.3)	-0.3 (-1.1, 0.2)	-0.3 (-1.0, 0.2)	-0.1 (-0.7, 0.4)	-0.2 (-0.8, 0.3)	-0.2 (-0.8, 0.2)	-0.3 (-1.2, 0.1)	-0.4 (-1.3, 0.2)	-0.4 (-1.2, 0.2)
	2030s	-0.3 (-1.0, 0.6)	-0.2 (-0.8, 0.7)	-0.3 (-1.1, 0.5)	0.3 (-0.4, 1.1)	0.3 (-0.5, 1.4)	0.2 (-0.6, 1.1)	-0.9 (-1.7, 0.1)	-0.6 (-1.2, -0.1)	-0.7 (-1.6, 0.0)
	2050s	-0.2 (-1.3, 1.0)	-0.1 (-1.1, 0.9)	0.4 (-0.8, 2.1)	0.4 (-0.6, 1.4)	0.4 (-0.6, 1.5)	1.1 (-0.2, 3.0)	-0.8 (-2.0, 0.7)	-0.7 (-1.6, 0.3)	-0.3 (-1.5, 1.2)
	2070s	-0.2 (-1.4, 0.8)	0.4 (-1.1, 2.0)	1.7 (-0.3, 4.2)	0.5 (-0.7, 1.4)	0.9 (-0.6, 2.6)	2.6 (0.4, 5.1)	-0.8 (-2.2, 0.2)	-0.2 (-1.6, 1.4)	0.8 (-0.9, 3.3)
	2090s	-0.3 (-1.6, 0.7)	0.6 (-1.0, 2.5)	3.9 (1.1, 7.8)	0.4 (-0.7, 1.4)	1.3 (-0.3, 3.2)	4.8 (1.4, 8.4)	-1.1 (-2.5, 0.1)	0.0 (-1.6, 1.9)	3.0 (0.8, 7.2)
Parkinson disease	2010s	0.7 (0.0, 1.4)	0.5 (-0.1, 1.1)	0.5 (-0.2, 1.2)	1.1 (0.2, 1.8)	0.9 (0.2, 1.7)	0.9 (0.1, 1.6)	0.3 (-0.3, 0.9)	0.1 (-0.4, 0.6)	0.2 (-0.5, 0.8)
	2030s	2.1 (0.6, 3.5)	1.8 (0.5, 3.5)	2.1 (0.8, 3.4)	3.0 (1.2, 4.6)	2.5 (0.9, 4.7)	3.0 (1.5, 4.3)	1.2 (0.0, 2.5)	1.0 (0.1, 2.4)	1.2 (0.1, 2.6)
	2050s	2.6 (0.8, 4.9)	2.8 (1.2, 5.0)	3.9 (2.0, 6.2)	3.6 (1.8, 6.0)	3.8 (2.1, 6.2)	5.2 (3.3, 7.2)	1.6 (-0.3, 3.8)	1.7 (0.3, 3.9)	2.6 (0.8, 5.1)
	2070s	2.7 (1.1, 4.7)	3.8 (1.7, 6.4)	6.5 (3.5, 10.0)	3.8 (2.2, 5.6)	4.7 (2.8, 7.3)	8.0 (4.8, 11.3)	1.6 (0.0, 3.7)	2.8 (0.6, 5.6)	4.9 (2.1, 8.6)
	2090s	2.6 (0.8, 5.0)	4.4 (2.2, 7.3)	9.9 (6.0, 14.9)	3.8 (2.1, 5.9)	5.7 (3.5, 8.3)	11.4 (7.5, 16.1)	1.4 (-0.4, 4.1)	3.2 (1.0, 6.3)	8.3 (4.6, 13.7)

Notes: The empirical 95% confidence intervals computed from Monte Carlo simulations (1,000 samples). SSP = Shared Socioeconomic Pathway.

Supplementary Table 10. The differences in net change of temperature-attributable number (means and empirical 95% confidence intervals) of neurodegenerative disease death in 2010-2090 compared with 1980–2009 under three climate change scenarios (SSP126, SSP245 and SSP585), classified by climatic zones and periods.

Diseases	Period	Nationwide			Temperate monsoon climate			Subtropical monsoon climate		
		SSP126	SSP245	SSP585	SSP126	SSP245	SSP585	SSP126	SSP245	SSP585
Neurodegenerative disease	2010s	-1881 (-3465, 211)	-1929 (-3714, -287)	-1986 (-4105, 4)	-27 (-448, 561)	-61 (-444, 317)	-77 (-647, 447)	-1854 (-3017, -350)	-1868 (-3270, -604)	-1909 (-3458, -444)
	2030s	-2187 (-6017, 1832)	-2441 (-4887, 5)	-983 (-4220, 3854)	371 (-853, 1351)	308 (-417, 1066)	876 (-79, 2012)	-2559 (-5164, 480)	-2749 (-4470, -1061)	-1859 (-4141, 1842)
	2050s	-1453 (-6785, 3451)	-824 (-5551, 3373)	3569 (-3639, 9664)	855 (-840, 2180)	1160 (-411, 2523)	2771 (349, 5354)	-2308 (-5945, 1272)	-1983 (-5140, 850)	799 (-3988, 4310)
	2070s	-981 (-6115, 5128)	1891 (-2412, 7314)	12768 (-1265, 26315)	1063 (-453, 2874)	2266 (553, 4208)	5881 (1318, 10393)	-2044 (-5662, 2255)	-375 (-2965, 3106)	6886 (-2583, 15922)
	2090s	-347 (-5927, 6684)	3808 (-3239, 11110)	28715 (1975, 55284)	1246 (-531, 3540)	2864 (438, 5354)	10803 (2459, 18664)	-1593 (-5396, 3144)	944 (-3677, 5756)	17911 (-484, 36620)
Dementia	2010s	-2066 (-3592, -219)	-2094 (-3837, -575)	-2145 (-3935, -459)	-217 (-579, 324)	-242 (-611, 82)	-258 (-736, 182)	-1849 (-3013, -543)	-1852 (-3226, -657)	-1887 (-3199, -642)
	2030s	-2909 (-6350, 822)	-3037 (-5372, -856)	-2005 (-5098, 2438)	-121 (-1168, 839)	-143 (-787, 525)	268 (-665, 1382)	-2788 (-5182, -17)	-2894 (-4585, -1381)	-2273 (-4433, 1055)
	2050s	-2499 (-6979, 1884)	-2159 (-6129, 1550)	1235 (-4607, 5902)	211 (-1229, 1437)	387 (-930, 1588)	1645 (-329, 3570)	-2710 (-5750, 447)	-2546 (-5199, -38)	-410 (-4278, 2332)
	2070s	-2214 (-6675, 3090)	-148 (-3811, 4484)	8374 (-3212, 18908)	341 (-987, 1965)	1228 (-169, 2850)	4036 (238, 7519)	-2555 (-5688, 1124)	-1375 (-3642, 1634)	4338 (-3450, 11388)
	2090s	-1661 (-6457, 4084)	1354 (-4526, 7474)	21314 (-870, 42652)	510 (-959, 2399)	1684 (-335, 3752)	8035 (1053, 14251)	-2171 (-5498, 1685)	-330 (-4191, 3722)	13279 (-1923, 28401)
Alzheimer disease	2010s	-539 (-1027, -82)	-537 (-1047, -114)	-549 (-1011, -113)	-35 (-112, 47)	-38 (-111, 32)	-38 (-116, 42)	-504 (-915, -130)	-499 (-936, -146)	-511 (-896, -154)
	2030s	-858 (-1772, 129)	-882 (-1611, -255)	-702 (-1644, 427)	-41 (-206, 127)	-49 (-188, 90)	-15 (-207, 177)	-817 (-1566, 2)	-833 (-1422, -345)	-688 (-1437, 250)
	2050s	-854 (-1966, 313)	-811 (-1943, 262)	-227 (-1632, 1067)	-27 (-235, 190)	-22 (-254, 212)	77 (-228, 396)	-828 (-1731, 123)	-789 (-1689, 50)	-304 (-1404, 671)
	2070s	-816 (-2038, 475)	-494 (-1702, 798)	983 (-1564, 3469)	-15 (-252, 233)	38 (-256, 329)	242 (-246, 756)	-801 (-1787, 242)	-532 (-1446, 469)	741 (-1317, 2714)
	2090s	-697 (-1914, 656)	-206 (-1795, 1397)	3343 (-1188, 7718)	0 (-228, 266)	73 (-268, 418)	534 (-201, 1285)	-698 (-1686, 390)	-279 (-1527, 979)	2809 (-986, 6433)
Non-Alzheimer dementias	2010s	-1520 (-2739, -55)	-1550 (-2902, -382)	-1588 (-3012, -255)	-182 (-523, 314)	-204 (-545, 97)	-220 (-653, 187)	-1339 (-2216, -369)	-1346 (-2357, -479)	-1369 (-2359, -442)
	2030s	-2042 (-4838, 893)	-2145 (-4047, -328)	-1295 (-3891, 2195)	-77 (-1050, 798)	-93 (-706, 540)	283 (-597, 1293)	-1965 (-3788, 95)	-2052 (-3341, -868)	-1578 (-3294, 902)
	2050s	-1637 (-5256, 1835)	-1339 (-4663, 1760)	1463 (-3354, 5664)	239 (-1092, 1366)	409 (-821, 1540)	1566 (-263, 3400)	-1876 (-4164, 469)	-1748 (-3842, 221)	-103 (-3091, 2264)
	2070s	-1391 (-5003, 2921)	352 (-2871, 4315)	7378 (-2187, 16512)	358 (-864, 1841)	1188 (-136, 2726)	3786 (264, 7090)	-1749 (-4138, 1080)	-837 (-2735, 1589)	3591 (-2451, 9422)
	2090s	-957 (-4844, 3765)	1562 (-3368, 6727)	17933 (-231, 35898)	512 (-846, 2248)	1609 (-283, 3571)	7485 (1024, 13327)	-1469 (-3998, 1517)	-46 (-3086, 3156)	10448 (-1255, 22570)
Parkinson disease	2010s	284 (-75, 753)	264 (-66, 671)	256 (-176, 748)	178 (34, 342)	171 (63, 313)	169 (50, 323)	106 (-109, 411)	93 (-129, 358)	87 (-226, 425)
	2030s	927 (123, 1667)	795 (190, 1541)	1230 (500, 2179)	450 (220, 671)	414 (233, 651)	542 (337, 847)	477 (-97, 995)	381 (-43, 890)	688 (162, 1333)
	2050s	1294 (177, 2301)	1607 (484, 2815)	2611 (1037, 4739)	584 (287, 876)	695 (401, 1084)	987 (576, 1631)	709 (-110, 1425)	912 (83, 1731)	1624 (461, 3108)
	2070s	1495 (418, 2575)	2332 (1005, 4105)	4672 (1969, 8438)	653 (358, 931)	919 (519, 1472)	1582 (896, 2626)	843 (59, 1644)	1413 (485, 2633)	3090 (1073, 5813)
	2090s	1562 (277, 3040)	2749 (1051, 4568)	7508 (2877, 13096)	659 (282, 1021)	1039 (556, 1562)	2310 (1161, 3713)	902 (-5, 2019)	1710 (494, 3006)	5198 (1716, 9383)

Notes: The empirical 95% confidence intervals computed from Monte Carlo simulations (1,000 samples). SSP = Shared Socioeconomic Pathway.

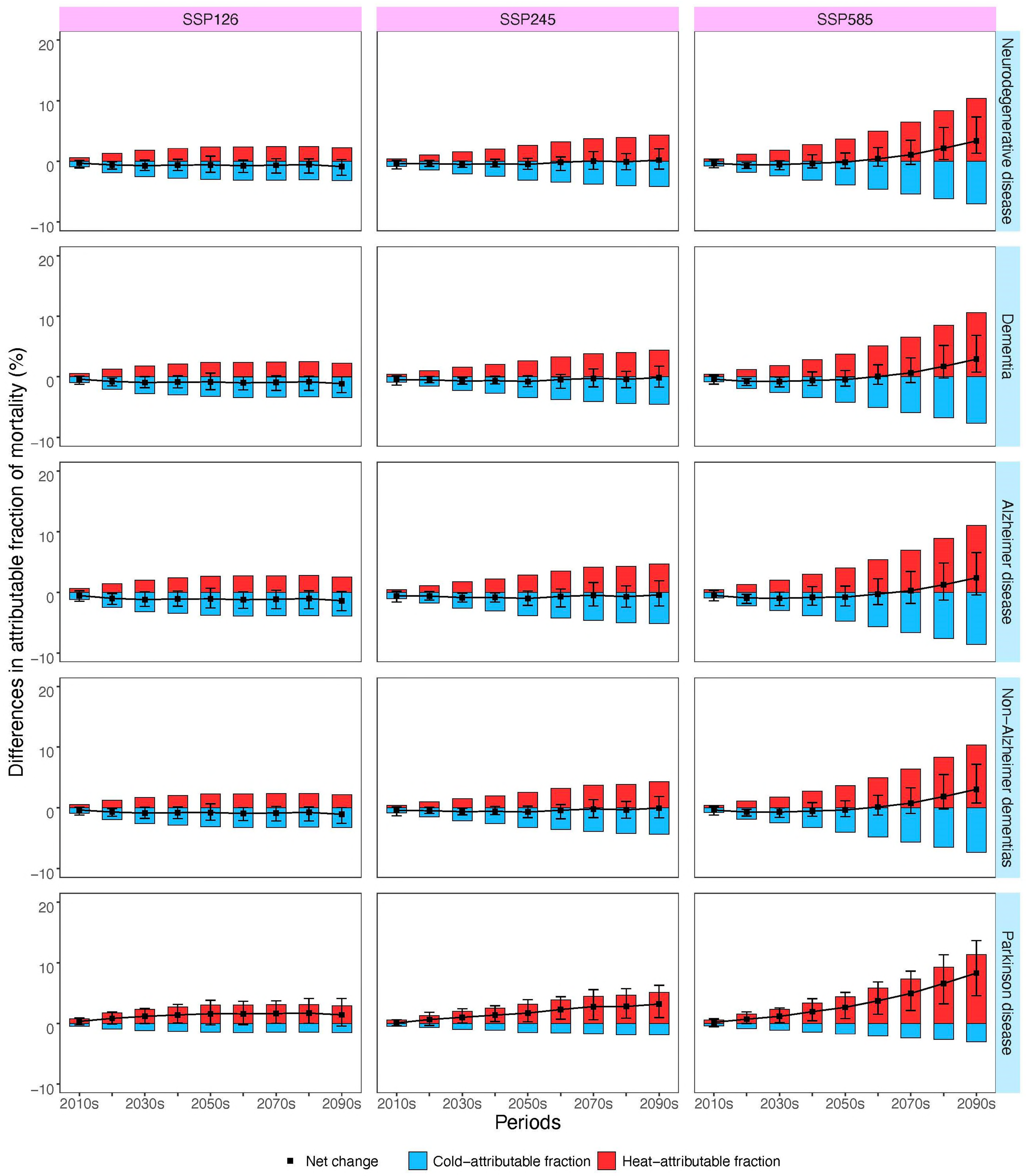

Supplementary Fig. 6. Differences in the projected fraction of neurodegenerative disease death associated with non-optimum temperatures in 2010–2090 compared with 1980–2009 under three climate change scenarios (SSP126, SSP245 and SSP585) in Subtropical monsoon climate zones of China. Estimates are reported as GCM-ensemble averages. The red bars represent the estimates of the heat-attributable fraction and the blue bars represent the estimates of the cold-attributable fraction. The black dots and vertical segments represent estimates of net differences and their 95% empirical CIs, respectively. SSP = Shared Socioeconomic Pathway. GCM = General climate models. Source data are provided as a Source Data file.

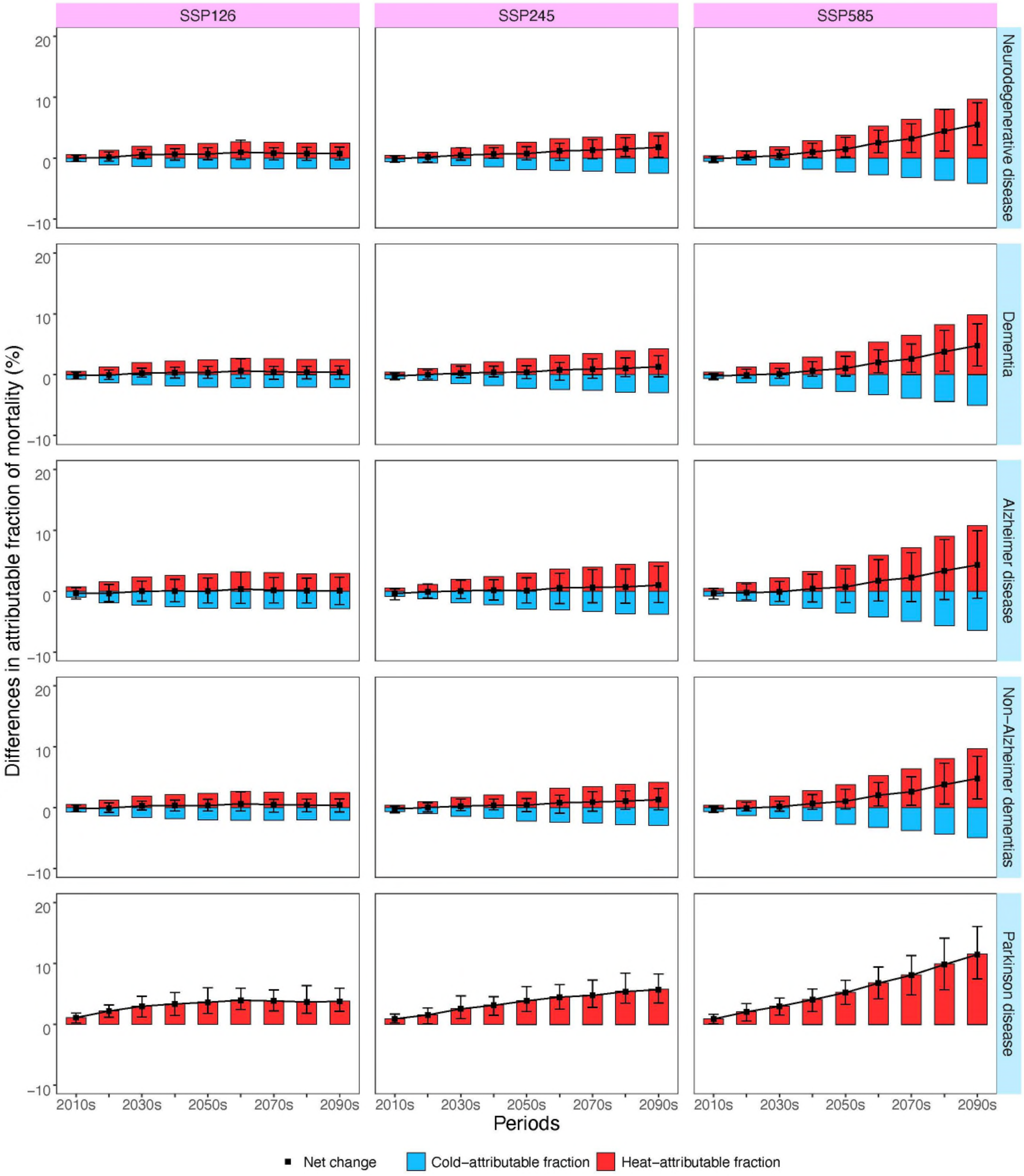

Supplementary Fig. 7. Differences in the projected fraction of neurodegenerative disease death associated with non-optimum temperatures in 2010–2090 compared with 1980–2009 under three climate change scenarios (SSP126, SSP245 and SSP585) in Temperate monsoon climate zones of China. Estimates are reported as GCM-ensemble averages. The red bars represent the estimates of the heat-attributable fraction and the blue bars represent the estimates of the cold-attributable fraction. The black dots and vertical segments represent estimates of net differences and their 95% empirical CIs, respectively. SSP = Shared Socioeconomic Pathway. GCM = General climate models. Source data are provided as a Source Data file.

Supplementary Table 11. Summary for the 10 general climate models of the NEX-GDDP-CMIP6 dataset.

General climate models[1]	Variant label
ACCESS-CM2	r1i1p1f1
ACCESS-ESM1-5	r1i1p1f1
BCC-CSM2-MR	r1i1p1f1
MIROC6	r1i1p1f1
MPI-ESM1-2-HR	r1i1p1f1
MPI-ESM1-2-LR	r1i1p1f1
MRI-ESM2-0	r1i1p1f1
NESM3	r1i1p1f1
NorESM2-LM	r1i1p1f1
NorESM2-MM	r1i1p1f1

Supplementary References

1. Thrasher B, Wang W, Michaelis A, Melton F, Lee T, Nemani R. NASA global daily downscaled projections, CMIP6. *Scientific Data* 2022; **9**(1): 262.
