# Supplementary material for: Temperature-related death burden of various neurodegenerative diseases under climate warming: a nationwide modelling study
Source: Nat Commun. 2023 Dec 12;14:8236. doi: 10.1038/s41467-023-44066-5 (PMC10716387; doi:10.1038/s41467-023-44066-5)
Supplement: Supplementary file 2 — Reporting Summary [file 41467_2023_44066_MOESM2_ESM.pdf]

nature portfolio

Corresponding author(s): Haidong Kan, Maigeng Zhou, Junwei Hao

Last updated by author(s): November 27, 2023

Reporting Summary

Nature Portfolio wishes to improve the reproducibility of the work that we publish. This form provides structure for consistency and transparency in reporting. For further information on Nature Portfolio policies, see our Editorial Policies and the Editorial Policy Checklist.

Please do not complete any field with "not applicable" or n/a. Refer to the help text for what text to use if an item is not relevant to your study.
For final submission: please carefully check your responses for accuracy; you will not be able to make changes later.

Statistics

For all statistical analyses, confirm that the following items are present in the figure legend, table legend, main text, or Methods section.

n/a	Confirmed	
☐	☒	The exact sample size (*n*) for each experimental group/condition, given as a discrete number and unit of measurement
☒	☐	A statement on whether measurements were taken from distinct samples or whether the same sample was measured repeatedly
☒	☐	The statistical test(s) used AND whether they are one- or two-sided<br>*Only common tests should be described solely by name; describe more complex techniques in the Methods section.*
☐	☒	A description of all covariates tested
☒	☐	A description of any assumptions or corrections, such as tests of normality and adjustment for multiple comparisons
☐	☒	A full description of the statistical parameters including central tendency (e.g. means) or other basic estimates (e.g. regression coefficient) AND variation (e.g. standard deviation) or associated estimates of uncertainty (e.g. confidence intervals)
☒	☐	For null hypothesis testing, the test statistic (e.g. *F*, *t*, *r*) with confidence intervals, effect sizes, degrees of freedom and *P* value noted<br>*Give P values as exact values whenever suitable.*
☒	☐	For Bayesian analysis, information on the choice of priors and Markov chain Monte Carlo settings
☒	☐	For hierarchical and complex designs, identification of the appropriate level for tests and full reporting of outcomes
☒	☐	Estimates of effect sizes (e.g. Cohen's *d*, Pearson's *r*), indicating how they were calculated

Our web collection on statistics for biologists contains articles on many of the points above.

Software and code

Policy information about availability of computer code

Data collection: All data were prepared using R software (version 3.6.1, R Project for Statistical Computing)

Data analysis: All statistical analyses were performed using R software (version 3.6.1, R Project for Statistical Computing) with the "dlnm" (2.4.7) and "survival" (3.2-7) package used for model construction and the "ggplot2" (3.4.2) package used for figure plotting. The custom code supporting the findings of this study is available upon request (send requests to kanh@fudan.edu.cn) .

For manuscripts utilizing custom algorithms or software that are central to the research but not yet described in published literature, software must be made available to editors and reviewers. We strongly encourage code deposition in a community repository (e.g. GitHub). See the Nature Portfolio guidelines for submitting code & software for further information.

Data

Policy information about availability of data

All manuscripts must include a data availability statement. This statement should provide the following information, where applicable:

- Accession codes, unique identifiers, or web links for publicly available datasets
- A description of any restrictions on data availability
- For clinical datasets or third party data, please ensure that the statement adheres to our policy

All data supporting the findings described in this manuscript are available in the article and in the Supplementary Information. The data generated in this study are available under restricted access for the identifiable nature of the data and data management requirements. Access can be obtained by contacting the corresponding author (kanh@fudan.edu.cn) and will be answered within 12 weeks. This study utilized death data from the China Cause of Death Reporting System (CDRS), which is not publicly available due to a restricted data use agreement with the national institute. Meteorological data were sourced from the fifth generation atmospheric reanalysis product (ERA5), accessible at https://cds.climate.copernicus.eu/cdsapp#!/search?type=dataset. The projected temperature data were obtained from the NASA Earth Exchange Global Daily Downscaled Projections dataset, available from https://www.nasa.gov/nex/gddp. Source data are provided with this paper.

Research involving human participants, their data, or biological material

Policy information about studies with human participants or human data. See also policy information about sex, gender (identity/presentation), and sexual orientation and race, ethnicity and racism.

Reporting on sex and gender	We collected individual death records of neurodegenerative diseases in all 2844 county-level administrative areas of China Mainland from 2013 to 2019. We also gathered information on demographics (e.g., gender, age, education level), date of death, and residential address.
Reporting on race, ethnicity, or other socially relevant groupings	We collected individual death records of neurodegenerative diseases in all 2844 county-level administrative areas of China Mainland from 2013 to 2019. We also gathered information on demographics (e.g., gender, age, education level), date of death, and residential address.
Population characteristics	More than 430,000 deaths from overall neurodegenerative diseases were evaluated in the study, including 375,776 deaths from dementias, 65,254 deaths from Alzheimer's disease, 310,522 deaths from non-Alzheimer dementias, and 51,428 deaths from Parkinson's disease. Among these, 92.2% were individuals aged 65 years or older, 53.5% were female, 91.6% had junior high school education or less. The subtropical monsoon zone and temperate monsoon zone accounted for 62.6% (N = 273,820) and 33.4% (N = 146,033) of overall neurodegenerative disease deaths, respectively.
Recruitment	Not applicable.
Ethics oversight	The study protocol was approved by the Institutional Review Board at the School of Public Health, Fudan University (IRB#2021-04-0889) with a waiver of informed consent.

Note that full information on the approval of the study protocol must also be provided in the manuscript.

Field-specific reporting

Please select the one below that is the best fit for your research. If you are not sure, read the appropriate sections before making your selection.

☐ Life sciences ☐ Behavioural & social sciences ☒ Ecological, evolutionary & environmental sciences

For a reference copy of the document with all sections, see nature.com/documents/nr-reporting-summary-flat.pdf

Ecological, evolutionary & environmental sciences study design

All studies must disclose on these points even when the disclosure is negative.

Study description	In this work, by virtue of the China's nationwide death registry, we conducted a large-scale, individual-level, case-crossover study to explore the associations between non-optimal temperatures and death from various neurodegenerative diseases. All individual deaths from neurodegenerative diseases in all 2,844 county-level administrative areas in mainland China from 2013 to 2019 were included. Additionally, we predicted the death burden in different climate zones under multiple climate change scenarios.
Research sample	We used death data from the China Cause of Death Reporting System (CDRS), a well-established system designed by the central government to collect information from all deaths occurring in China Mainland. The data from this system are widely used by the central government to produce official mortality statistics for informing health policy, as well as being a reliable data source for scientific research. More than 430,000 deaths from overall neurodegenerative diseases were evaluated in the study, including 375,776 deaths from dementias, 65,254 deaths from Alzheimer's disease, 310,522 deaths from non-Alzheimer dementias, and 51,428 deaths from Parkinson's disease. Among these, 92.2% were individuals aged 65 years or older, 53.5% were female, 91.6% had junior high school education or less. The subtropical monsoon zone and temperate monsoon zone accounted for 62.6% (N = 273,820) and 33.4% (N = 146,033) of overall neurodegenerative disease deaths, respectively.
Sampling strategy	All individual deaths from neurodegenerative diseases in all 2,844 county-level administrative areas in mainland China from 2013 to 2019 were included. As per the 10th edition of International Classification of Diseases, we derived the data of overall neurodegenerative diseases, dementia, Alzheimer's disease, non-Alzheimer's dementia, and Parkinson's disease according to the underlying cause of death

Data collection	We used death data from the China Cause of Death Reporting System (CDRS), a well-established system designed by the central government to collect information from all deaths occurring in China Mainland. The data collection process adhered to stringent protocols, standard procedures, and meticulous quality control measures. The data from this system are widely used by the central government to produce official mortality statistics for informing health policy, as well as being a reliable data source for scientific research.We collected individual death records of neurodegenerative diseases in all 2844 county-level administrative areas of China Mainland from 2013 to 2019. As per the 10th edition of International Classification of Diseases, we derived the data of overall neurodegenerative diseases, dementia, Alzheimer's disease, non-Alzheimer's dementia, and Parkinson's disease according to the underlying cause of death. We also gathered information on demographics (e.g., gender, age, education level), date of death, and residential address. Meteorological data were sourced from the fifth generation atmospheric reanalysis product (ERA5). The projected temperature data were obtained from the NASA Earth Exchange Global Daily Downscaled Projections dataset.
Timing and spatial scale	We collected individual death records of neurodegenerative diseases in all 2844 county-level administrative areas of China Mainland from January 1, 2013 to December 31, 2019. This analysis was conducted at both the national level and within different climatic zones, including the subtropical monsoon zone, temperate monsoon zone, temperate continental zone, tropical monsoon zone, and highland alpine zone. The meteorological data were obtained from the ERA5 reanalysis product developed by the European Centre for Medium-Range Weather Forecasts with 0.1° × 0.1° spatial and hourly temporal resolutions. The projected temperature data were obtained from the NASA Earth Exchange Global Daily Downscaled Projections dataset. The downscaled products are produced using daily variants of the monthly bias correction/spatial decomposition method, with a horizontal resolution of ¼ degree. We extracted simulated daily temperature series for each county for the period 1980-2099.
Data exclusions	No data were excluded from the analyses
Reproducibility	We repeated the experiment three times before submission as per the initial experimental code and data, and all attempts to repeat the experiment were successful.
Randomization	This is an individual-level, time-stratified, case-crossover study. This kind of design could minimize the confounding effect of all individual-level, time-invariant risk factors through a self-matching strategy and automatically excludes temporal trends (e.g., seasonality) by selecting controls within a month.
Blinding	This study is a case cross-over design. Blinding was not applicable to the self-control data in this study

Did the study involve field work? ☐ Yes ☒ No

Reporting for specific materials, systems and methods

We require information from authors about some types of materials, experimental systems and methods used in many studies. Here, indicate whether each material, system or method listed is relevant to your study. If you are not sure if a list item applies to your research, read the appropriate section before selecting a response.

Materials & experimental systems

n/a	Involved in the study
☒	☐ Antibodies
☒	☐ Eukaryotic cell lines
☒	☐ Palaeontology and archaeology
☒	☐ Animals and other organisms
☒	☐ Clinical data
☒	☐ Dual use research of concern
☒	☐ Plants

Methods

n/a	Involved in the study
☒	☐ ChIP-seq
☒	☐ Flow cytometry
☒	☐ MRI-based neuroimaging
